# Supplementary material for: Bacteria loaded with glucose polymer and photosensitive ICG silicon-nanoparticles for glioblastoma photothermal immunotherapy
Source: Nat Commun. 2022 Sep 1;13:5127. doi: 10.1038/s41467-022-32837-5 (PMC9433534; doi:10.1038/s41467-022-32837-5)
Supplement: Supplementary file 1 — Supplementary Information [file 41467_2022_32837_MOESM1_ESM.pdf]

Bacteria loaded with glucose polymer and photosentisitve ICG silicon-nanoparticles for glioblastoma photothermal immunotherapy

Rong Sun[1, ‡], Mingzhu Liu[1, ‡], Jianping Lu[1, ‡], Binbin Chu[1], Yunmin Yang[1], Bin Song[1], Houyu Wang[1, *] & Yao He[1, *]

[1]Suzhou Key Laboratory of Nanotechnology and Biomedicine, Institute of Functional Nano & Soft Materials & Collaborative Innovation Center of Suzhou Nano Science and Technology (NANO-CIC), Soochow University, Suzhou 215123, China

*Corresponding authors. Email: houyuwang@suda.edu.cn; yaohe@suda.edu.cn

‡These authors contributed equally to this work.

Inventory of Supplementary Information

- Supplementary Figures S1-S20
- Supplementary Tables 1-4
- Supplementary Notes 1-2

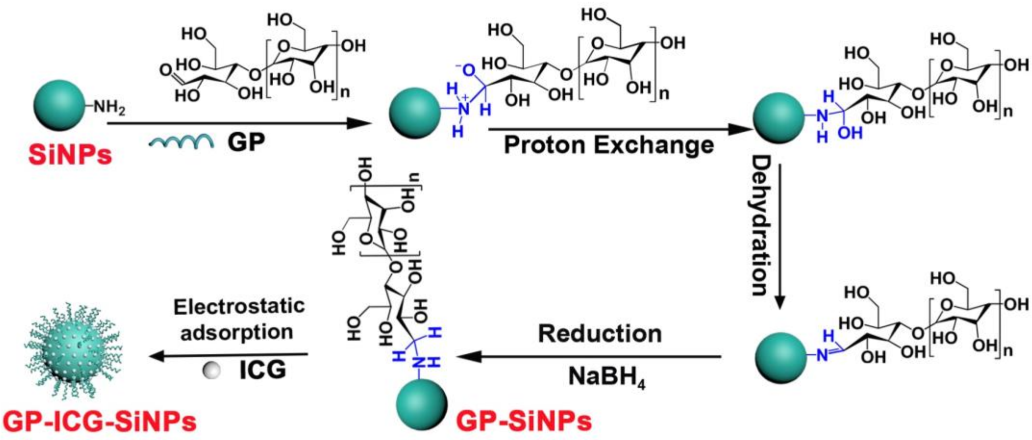

Supplementary Fig. 1. Schematic diagram of the synthesis of nanoagents GP-ICG-SiNPs. The GP molecules (e.g, *poly[4-O-(α-D-glucopyranosyl)-D-glucopyranose]*) are firstly conjugated to the SiNPs surface based on the Schiff base reaction between the aldehyde groups of GP and the amino groups terminated SiNPs. After that, the ICG molecules are loaded on GP-SiNPs through electrostatic adsorption.

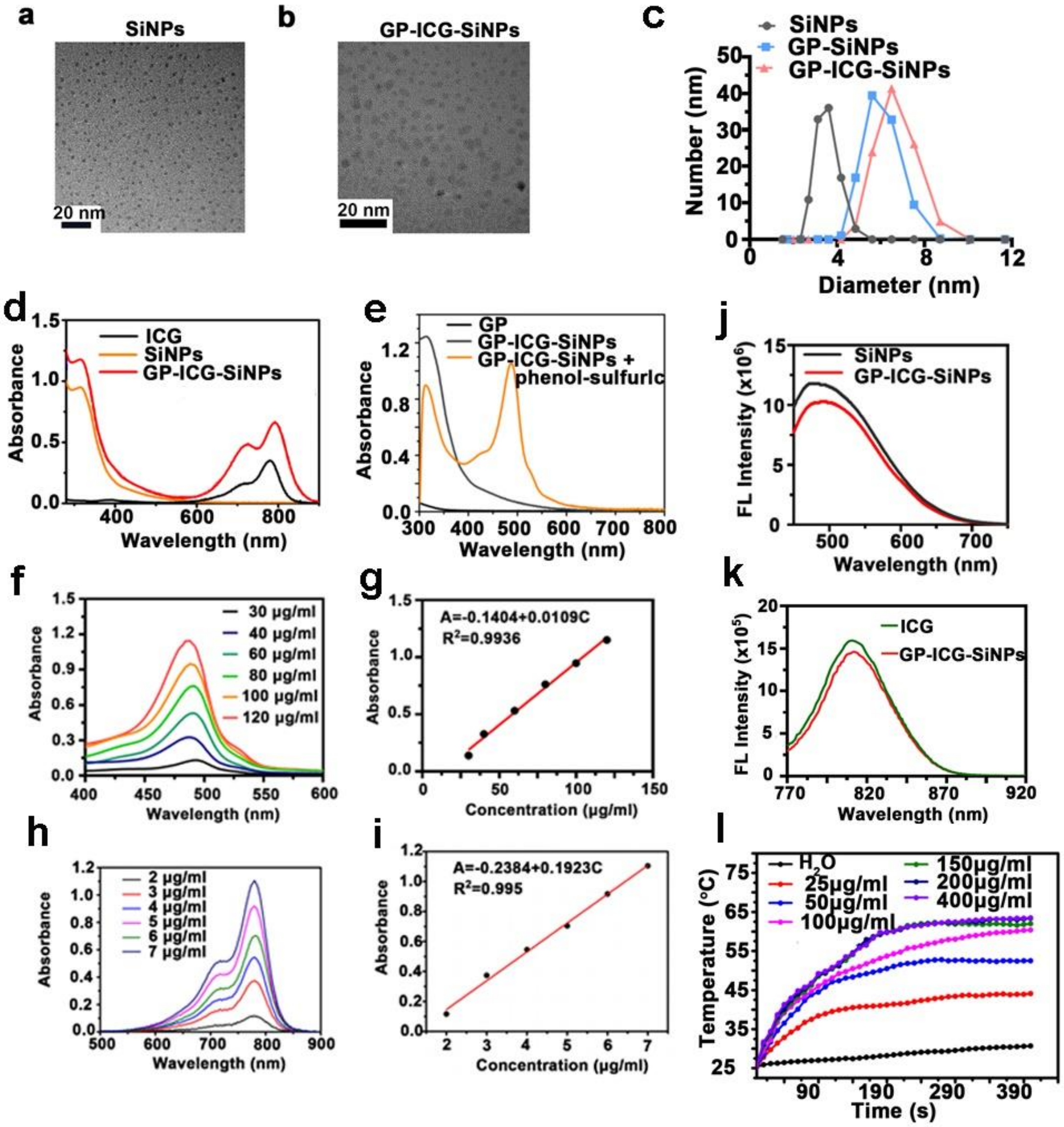

Supplementary Fig. 2. Characterizations of the nanoagents. a, TEM image of SiNPs. **b,** TEM image of GP-ICG-SiNPs. All imaging experiments were repeated three times with similar results. Scale bars: 20 nm. GP-ICG-SiNPs appear as spherical particles with a narrow size distribution of ~3.2 nm, which is slightly larger than that of bare SiNPs (e.g., ~2.7 nm). **c,** The DLS data of SiNPs, GP-SiNPs and GP-ICG-SiNPs. The hydrodynamic diameter of GP-ICG-SiNPs is ~5.6 nm, also larger than the hydrodynamic diameter of bare SiNPs (~3.0 nm). **d,** The UV-vis absorbance of ICG, SiNPs and GP-ICG-SiNPs. **e,** The UV-vis absorbance of GP, GP-ICG-SiNPs, and GP-ICG-SiNPs treated with the phenol-sulfuric acid. **f-i**, UV-vis absorption spectra of GP with various concentrations ranged from 30 to 120 μg/mL treated by the same amount

of phenol-sulfuric acid **(f)** and corresponding calibration curve **(g)** and UV-vis absorption spectra of ICG with various concentrations ranged from 0 to 10 μg/mL **(h)** and corresponding calibration curve **(i)**. **j,** The PL spectra of SiNPs and GP-ICG-SiNPs under the excitation of 405 nm. **k,** The PL spectra of ICG and GP-ICG-SiNPs under the excitation of 780 nm. **l**, Photothermal heating curves of GP-ICG-SiNPs containing various ICG concentrations under the NIR laser (808 nm, 1 W/cm^2) irradiation. The temperature of GP-ICG-SiNPs solutions can be enhanced by 30 °C during 300-sec 808-nm laser exposure when the loading concentration of ICG is or more than 150 μg/mL.

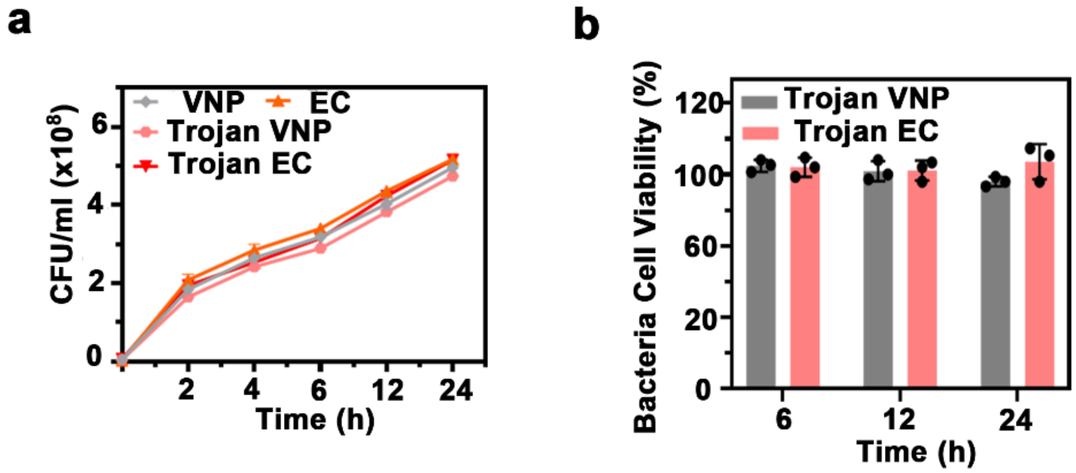

Supplementary Fig. 3. The effects of the internalized nanoagents on the growth and activity of host bacteria. a, The growth curves of bacteria (VNP and EC) and Trojan bacteria (Trojan VNP and Trojan EC) during 24-h culturing. **b,** The bacteria cell viability of bacteria (VNP and EC) and Trojan bacteria (Trojan VNP and Trojan EC) during 24-h culturing. All error bars represent the standard deviation determined from three independent assays. All data are presented as means ± SD.

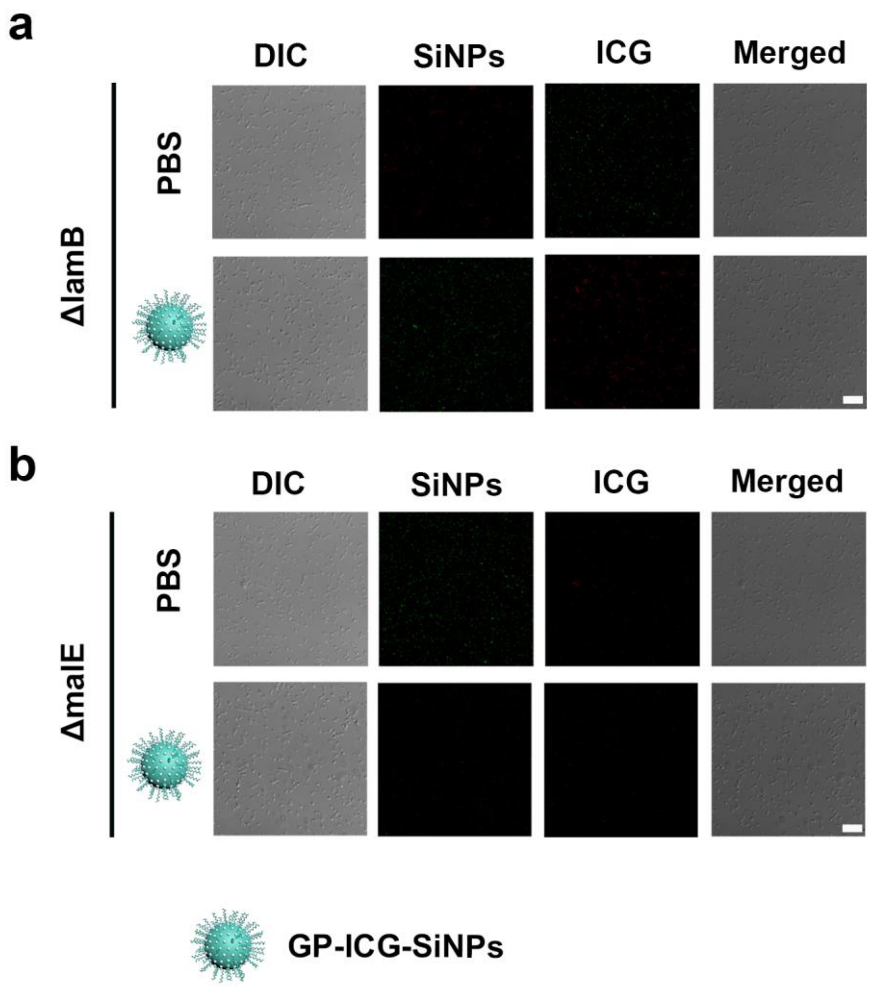

Supplementary Fig. 4. Confocal fluorescence images of bacteria mutants of ΔlamB (a) and ΔmalE (b) after incubation with GP-ICG-SiNPs at 37 °C for 2 h, followed by washing with PBS buffer several times. Scale bars: 25 μm. All imaging experiments were repeated three times with similar results.

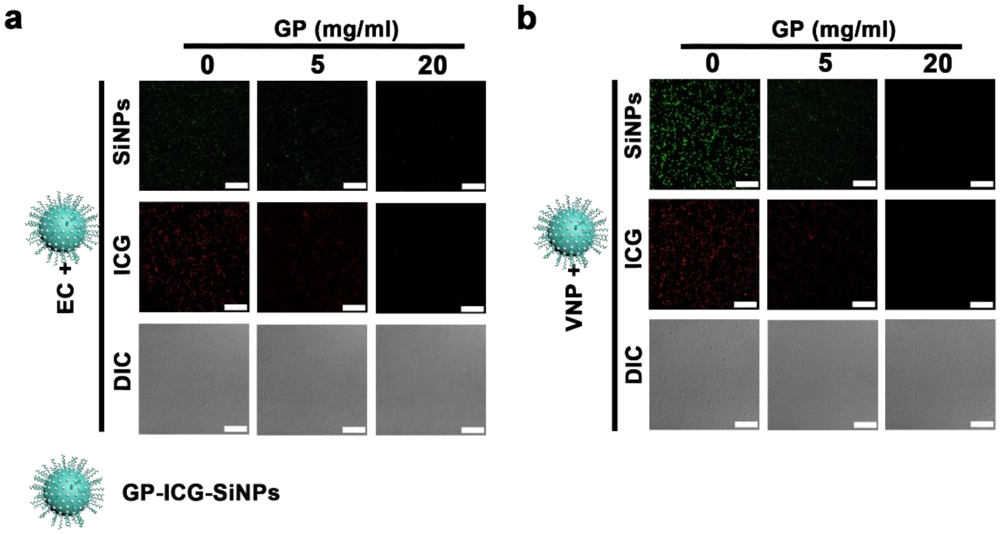

Supplementary Fig. 5. Fluorescent confocal images of nanoagents-treated with bacteria after treatments of free GP molecules with various concentrations. a, Confocal fluorescence images of EC treated with GP at 0, 5, and 20 mg/ml and incubated with 10 mg/mL GP-ICG-SiNPs for 2 h at 37 ºC. Scale bars: 20 μm. **b,** Confocal fluorescence images of VNP treated with GP at 0, 5, and 20 mg/ml and incubated with 10 mg/mL GP-ICG-SiNPs for 2 h at 37 ºC. Scale bars: 20 μm. All imaging experiments were repeated three times with similar results.

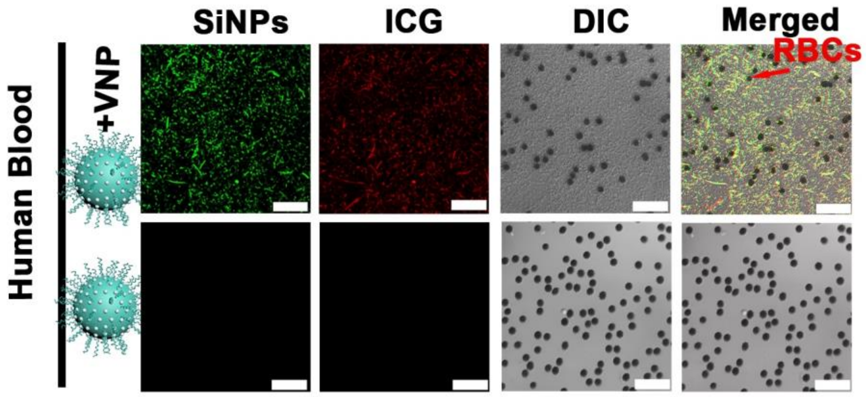

Supplementary Fig. 6. CLSM images of the mixture of human blood and VNP after incubation with GP-ICG-SiNPs. Arrows indicate red blood cells (RBCs). Scale bar: 25 μm. The VNP were incubated with the synthesized nanoagents ([SiNPs] = 12 mg/mL, [ICG] = 600 μg/mL) at 37 ºC for 2 h. After incubation, the treated bacteria were rinsed with PBS buffer for several times. The bacterial cell concentration is ~1.0 $\times 10^7$ CFU. All imaging experiments were repeated three times with similar results.

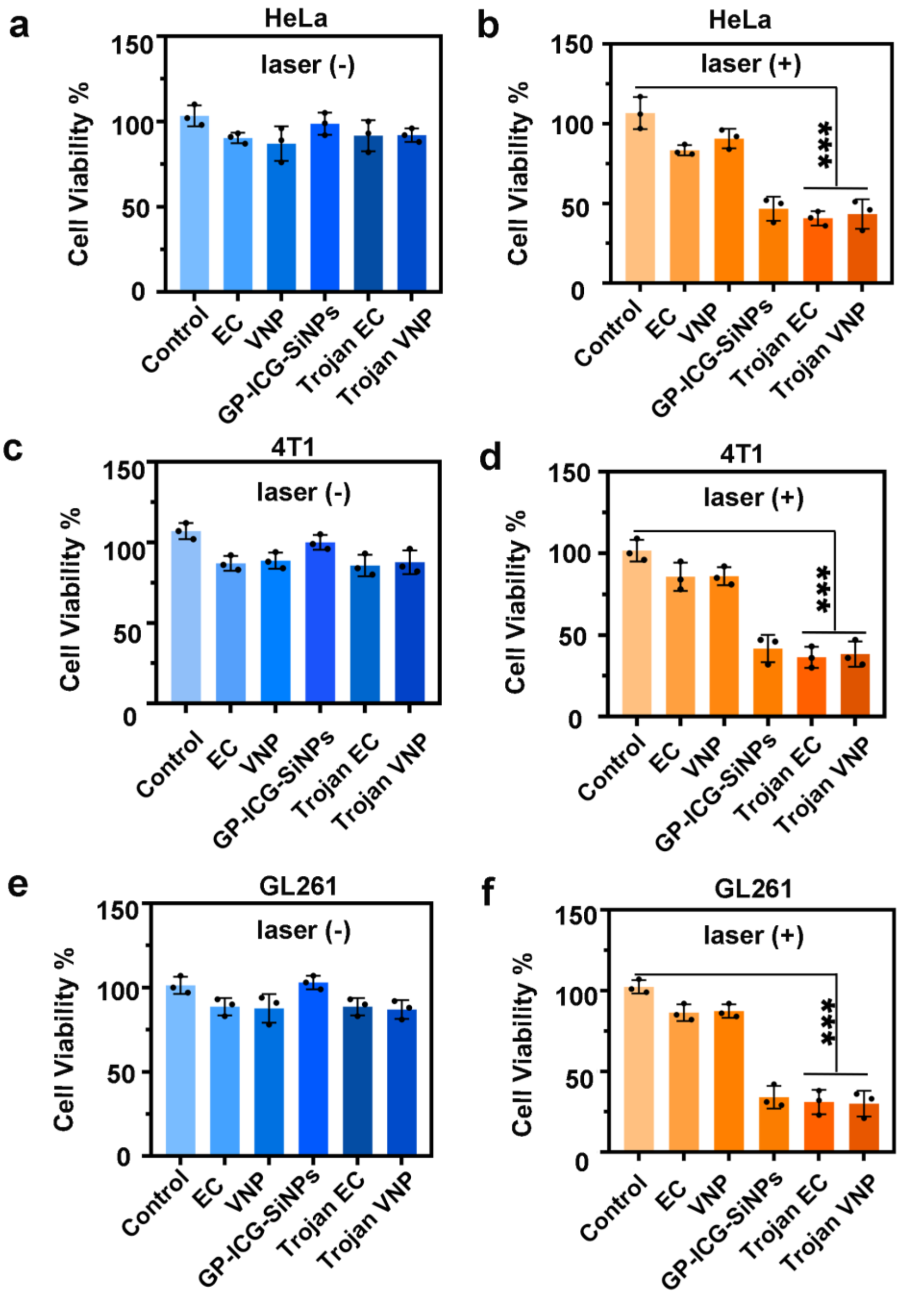

Supplementary Fig. 7. The viability of HeLa cells treated with EC, VNP, GP-ICG-SiNPs, Trojan EC and Trojan VNP with (**a**) or without laser irradiation (**b**) for 5 minutes (808 nm, 1.2 W/cm^2). Data are presented as mean values +/- SD (n = 3). The viability of 4T1 cells treated with EC, VNP, GP-ICG-SiNPs, Trojan EC and Trojan VNP with (**c**) or without laser irradiation (**d**) for 5 minutes (808 nm, 1.2 W/cm^2). Data are presented as mean values +/- SD (n = 3). The viability of GL261 cells treated with EC, VNP, GP-ICG-SiNPs, Trojan EC and Trojan VNP with (**e**) or without laser irradiation (**f**) for 5

minutes (808 nm, 1.2 W/cm^2). ***p=1.3×10^{-4} for Control vs. Trojan EC, ***p=1.5×10^{-4} for Control vs. Trojan VNP (**b**), ***p=4.80×10^{-4} for Control vs. Trojan EC, ***p=1.30×10^{-3} for Control vs. Trojan VNP (**d**), ***p=2.60×10^{-4} for Control vs. Trojan EC, ***p=4.20×10^{-4} for Control vs. Trojan VNP (**f**). Data are presented as mean values +/- SD (n = 3). Statistical significance was calculated *via* one-way analysis of variance (ANOVA) with a Tukey post-hoc test.

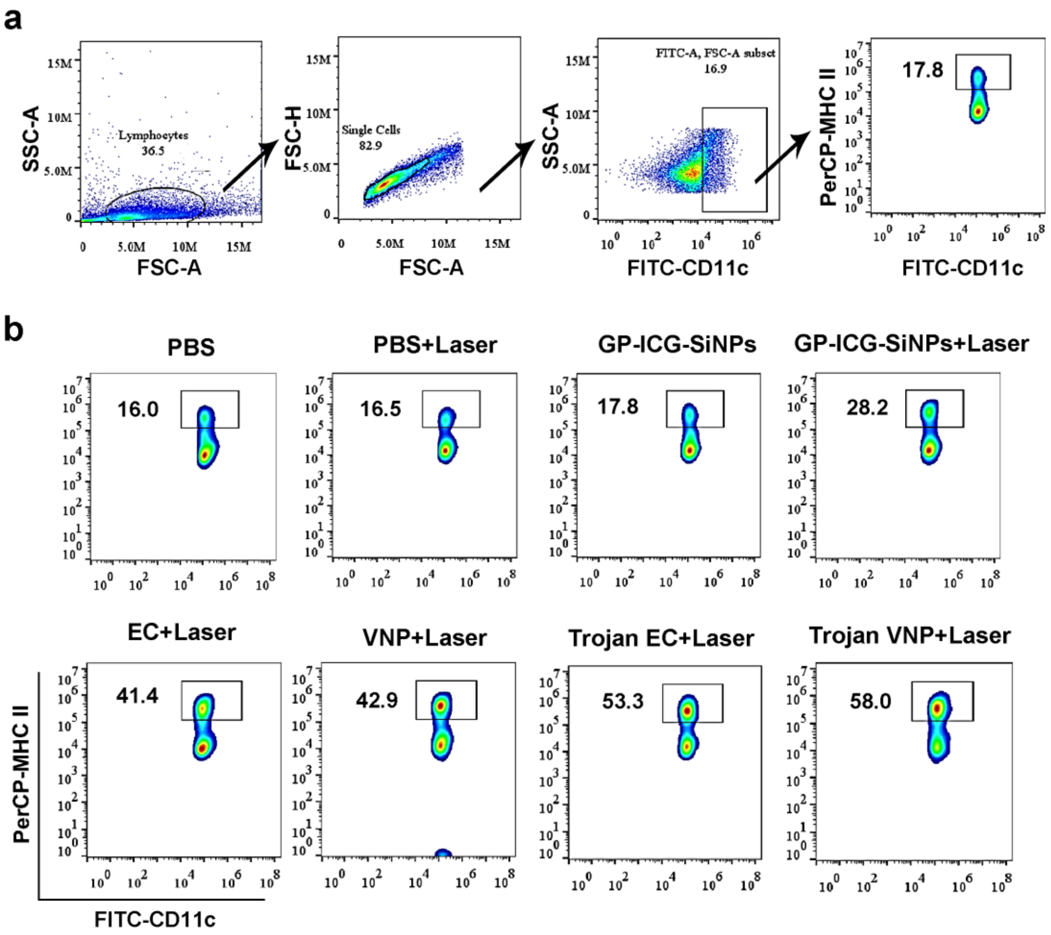

Supplementary Fig. 8. Gating strategy to determine the percent of matured DC cells ($CD11c^{+}$ MHC II^{+}) (a), and representative flow cytometric analysis of matured DC cells (b) post different treatments as indicated in the transwell system.

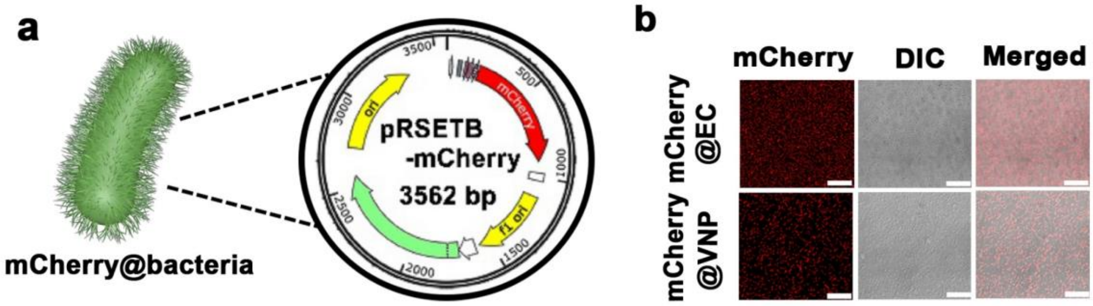

Supplementary Fig. 9. The construction of bacteria transformants. a, A scheme illustrating the plasmid of pRSETB-mCherry. **b,** Confocal fluorescence images of constructed mCherry@EC and mCherry@VNP expressing mCherry protein. Scale bars: 25 μm. The confocal images show the red fluorescence signals of mCherry (λ_{ex} = 543 nm, λ_{em} = 580-650 nm) from the EC or VNP, suggesting the successful construction of bacteria transformants. All imaging experiments were repeated three times with similar results.

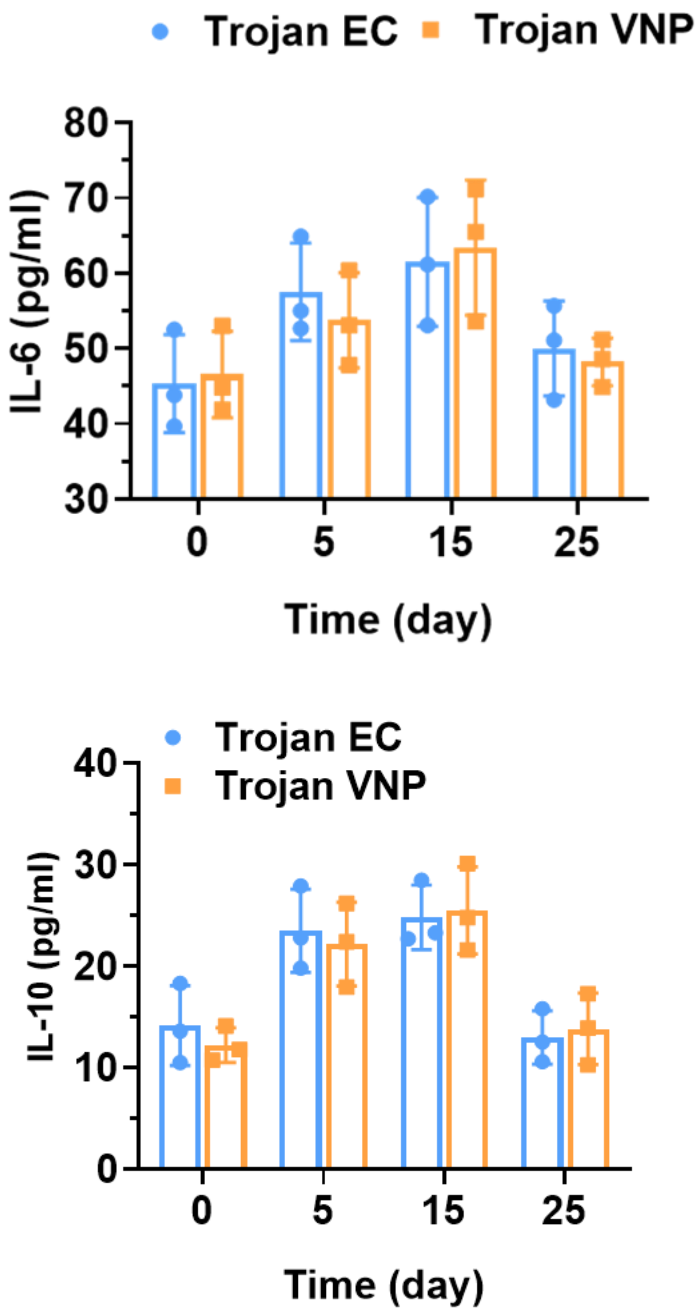

Supplementary Fig. 10. IL-6 and IL-10 levels in cerebrospinal fluids of tumour-bearing mice intravenously injected with Trojan EC or Trojan VNP at the dose of 1.0×10^{7} CFU per mouse at 0, 5, 15 and 25 d post injection. Error bars represent the standard deviation obtained from three independent measurements.

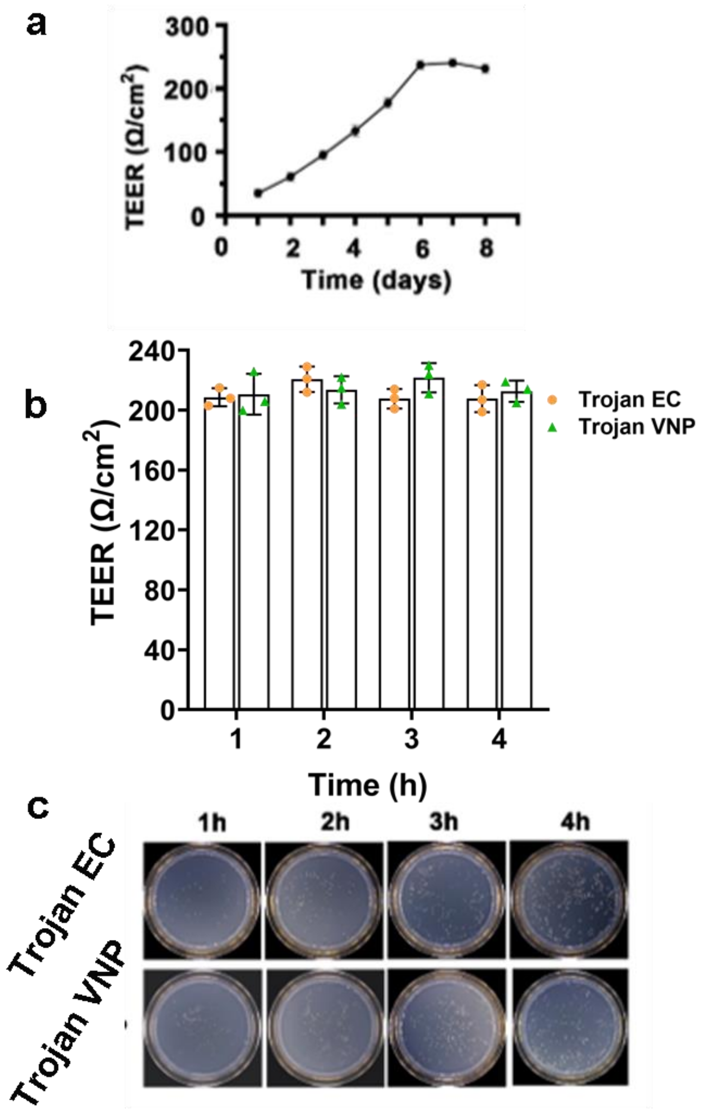

Supplementary Fig. 11. Evaluation of the constructed HBMEC model. a, Change in the TEER value of the HBMEC cell monolayer during culture. When the TEER value is 150-300 Ω/cm^2, the constructed HBMEC cell monolayer can be used to study BBB penetration. **b,** The TEER of the constructed HBMEC BBB model after co-incubating with Trojan EC or Trojan VNP at 1, 2, 3, 4 h. **c,** The colony growth of the medium taken from the lower chamber of the transwell on the LB plate at 1h, 2h, 3h, 4 h. Error bars represent the standard deviation obtained from three independent measurements.

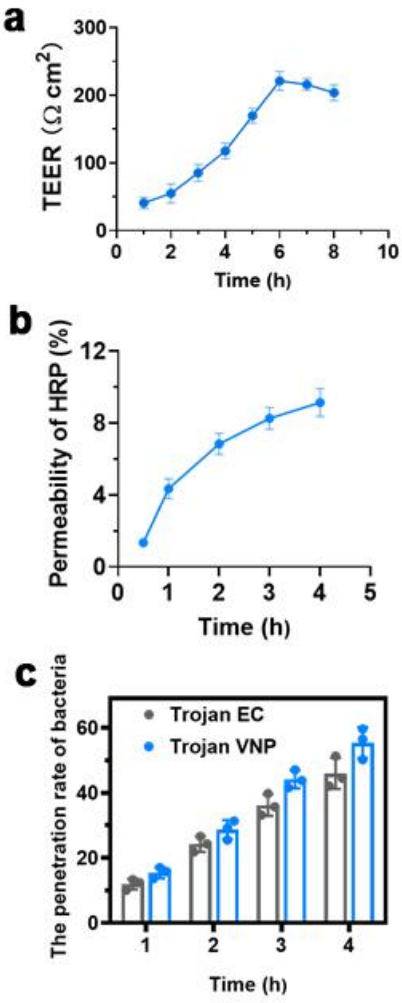

Supplementary Fig.12 Change in the TEER value of bEnd.3 cells-based BBB model during culture (a), the penetration of horseradish peroxidase (HRP) in the constructed BBB model (b), and the corresponding penetration rates of Trojan EC or Trojan VNP at 1, 2, 3 and 4h in the bEnd.3 cells-based BBB model (mean ± SD, n = 3) (c).

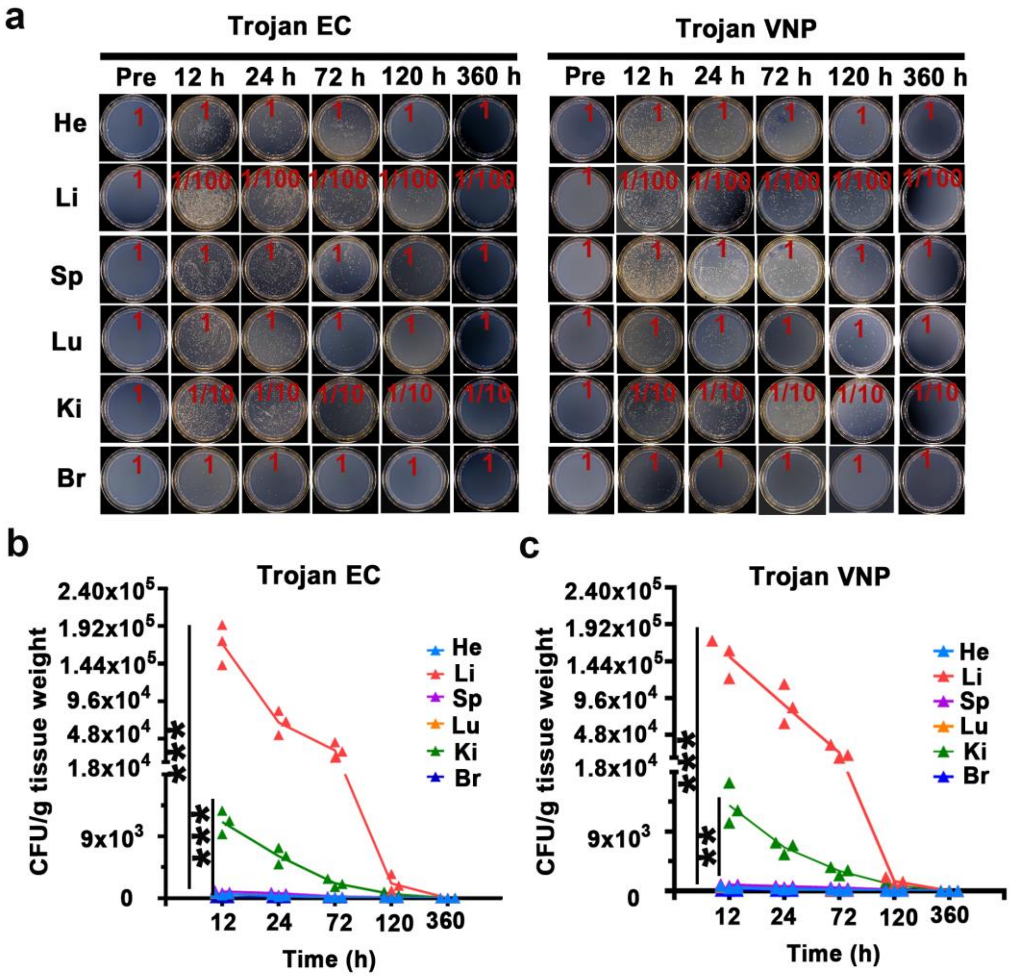

Supplementary Fig. 13 Homogenates of major organs of healthy mice after intravenous injection with Trojan EC (left) and Trojan VNP (right) for 12, 24, 72 120 and 360 h cultured on the solid LB agar (n=3) (**a**) and corresponding quantification of bacterial colonization in mCherry@EC group (**b**) and mCherry@VNP group (**c**). ***p=3.50×10^{-5} for liver vs. brain, ***p=3.4×10^{-4} for kidney vs. brain (**b**), ***p=5.10×10^{-4} for liver vs. brain, **p=1.9×10^{-3} for kidney vs. brain (**c**). Statistical significance was calculated *via* one-way analysis of variance (ANOVA) with a Tukey post-hoc test. Data are presented as mean values +/- SD (n = 3).

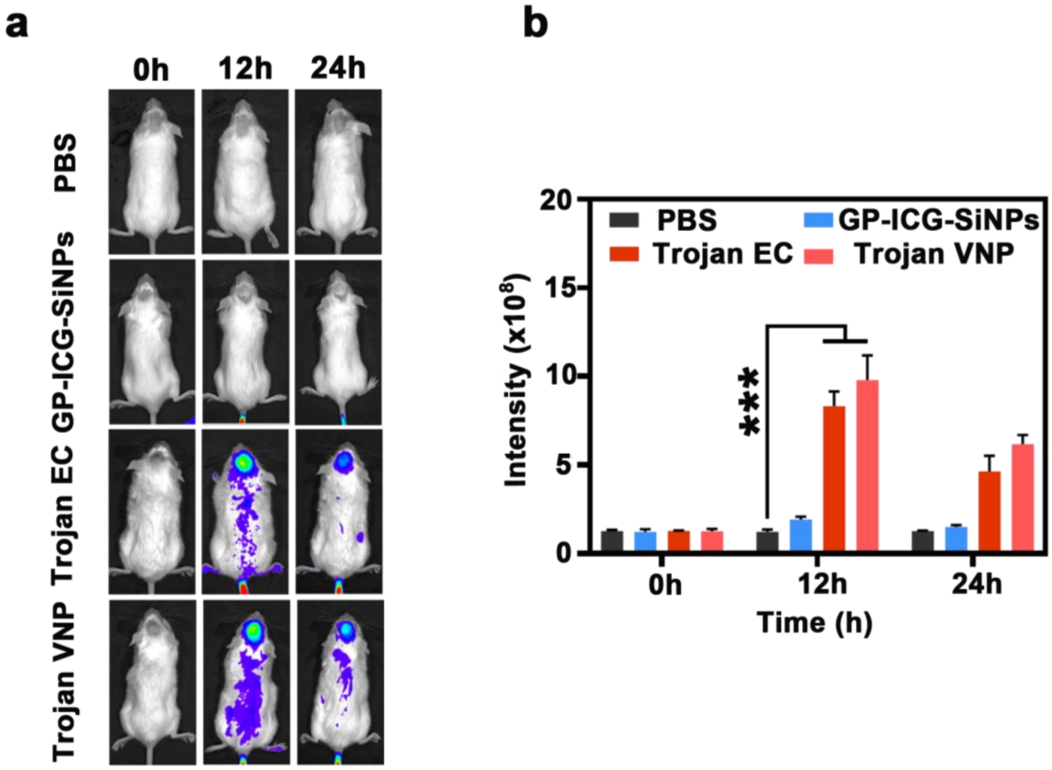

Supplementary Fig. 14. ***In vivo*** **fluorescence imaging of in situ tumor-bearing mice.** **a,** *In vivo* real-time fluorescence imaging of tumor-bearing mice at different time points under different drug treatments. **b,** Fluorescence quantitative analysis of GBM was performed at different time points. ***p=6.90×10^{-3} for PBS vs. Trojan EC, ***p=1.30×10^{-3} for PBS vs. Trojan VNP. All error bars represent the standard deviation determined from three independent assays. Statistical significance was calculated *via* one-way analysis of variance (ANOVA) with a Tukey post-hoc test. Data are presented as mean values +/- SD (n = 3).

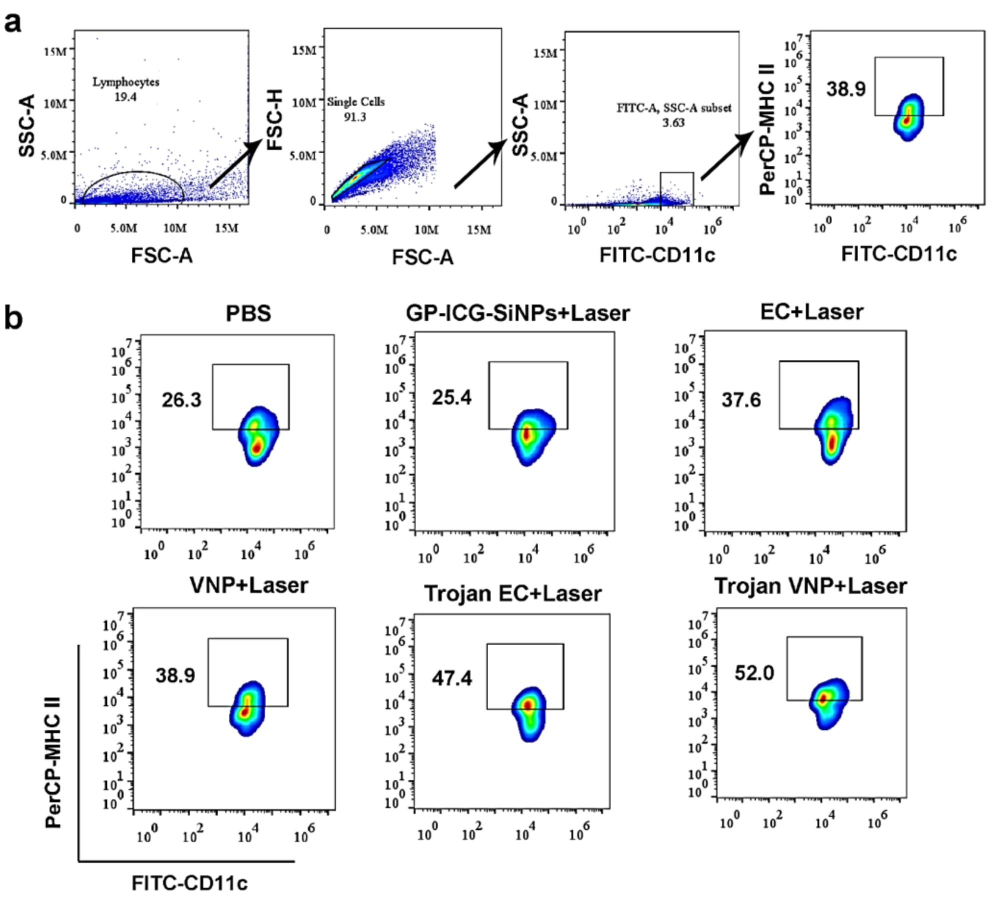

Supplementary Fig. 15. Gating strategy to determine the percent of matured DC cells ($CD11c^{+}$ MHC II^{+}) (**a**), and representative flow cytometric analysis of matured DC cells (**b**) in the lymph nodes of mice post different treatments as indicated.

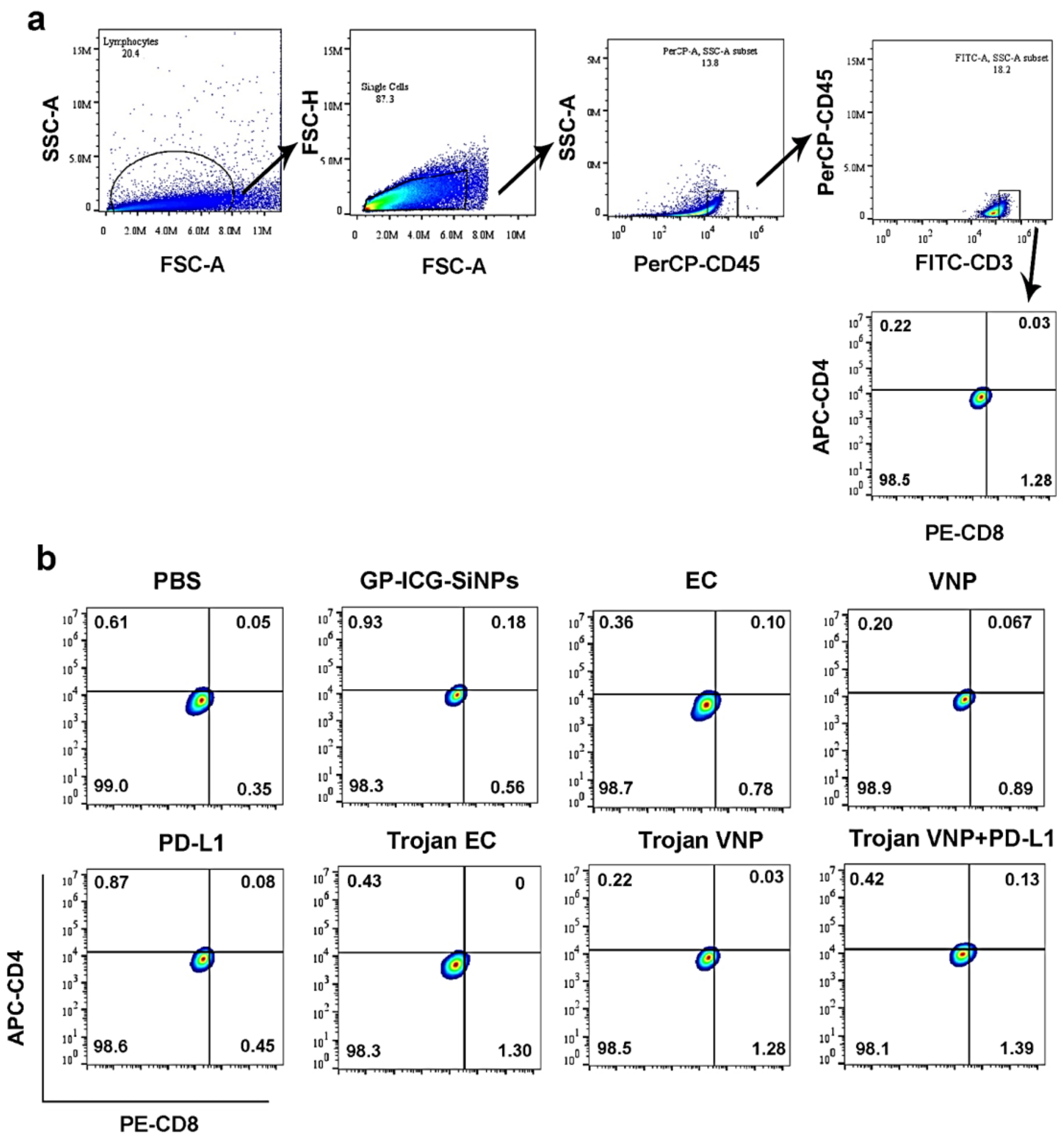

Supplementary Fig. 16. Gating strategy to determine the percent of $CD8^+$ T cells ($CD45^+CD3^+CD8^+$ $CD4^+$) (a), and representative flow cytometric analysis of $CD8^+$ T cells (b) in the tumours of these mice post different treatments as indicated.

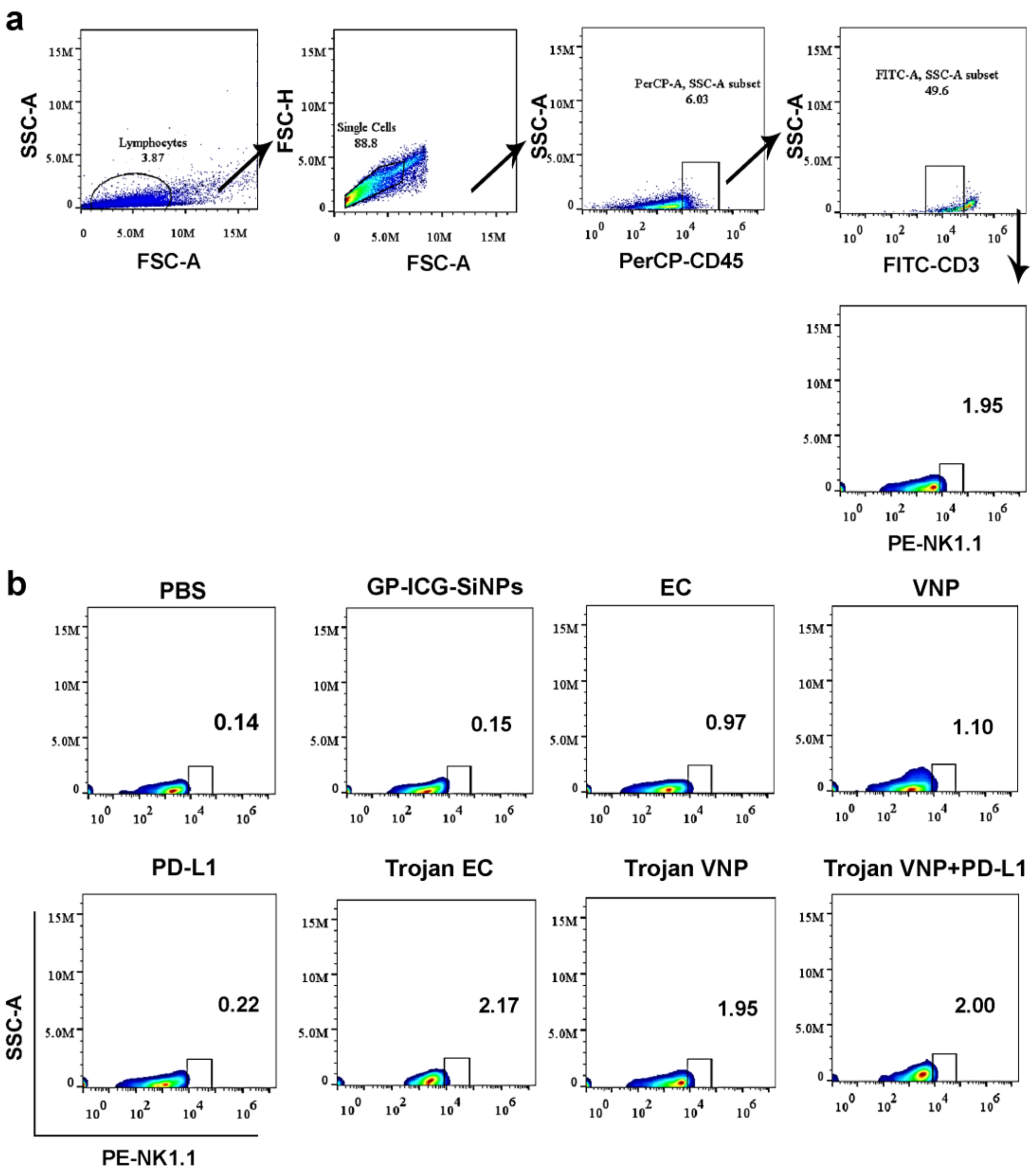

Supplementary Fig. 17. Gating strategy to determine the percent of NK cells ($CD45^{+}CD3^{+}NK1.1^{+}$) (a), and representative flow cytometric analysis of NK cells (b) in the tumours of these mice post different treatments as indicated.

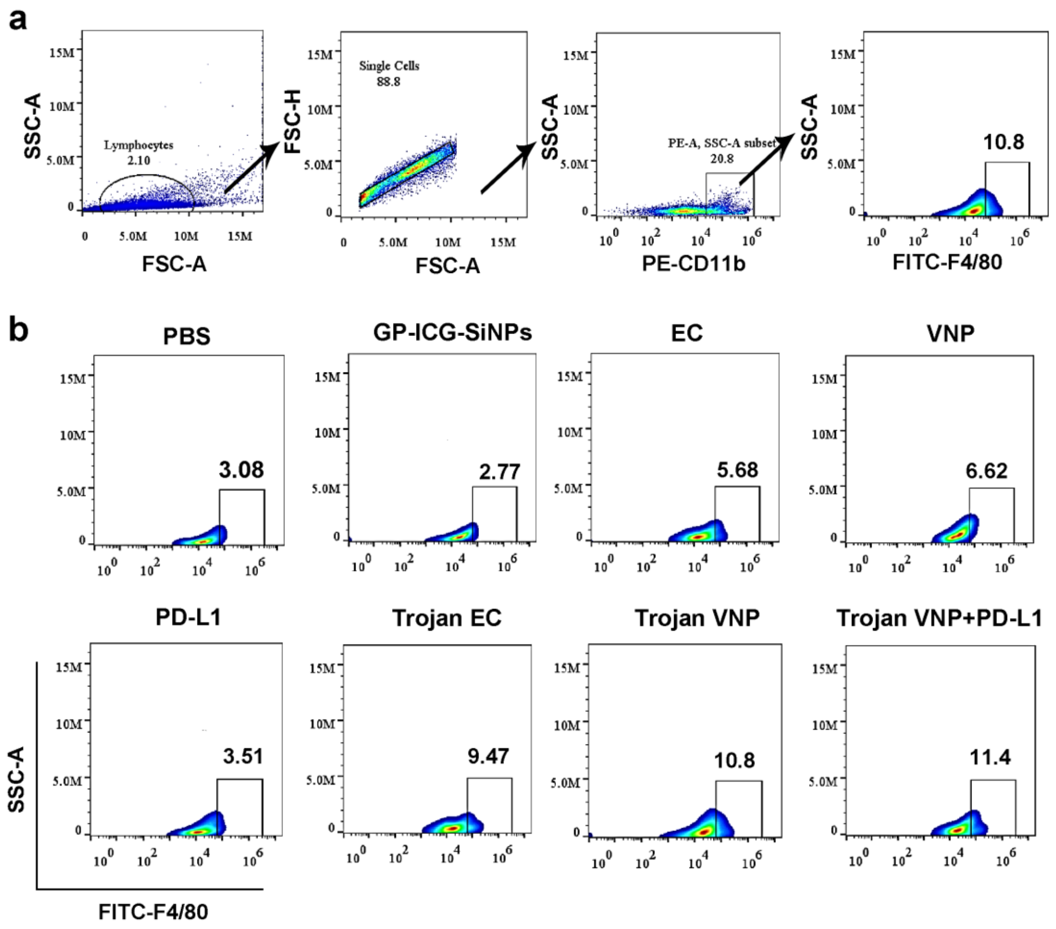

Supplementary Fig. 18 Gating strategy to determine the percent of macrophage cells ($CD11b^+$, $F4180^+$) (a), and representative flow cytometric analysis of macrophages (b) in the tumors of these mice post different treatments as indicated.

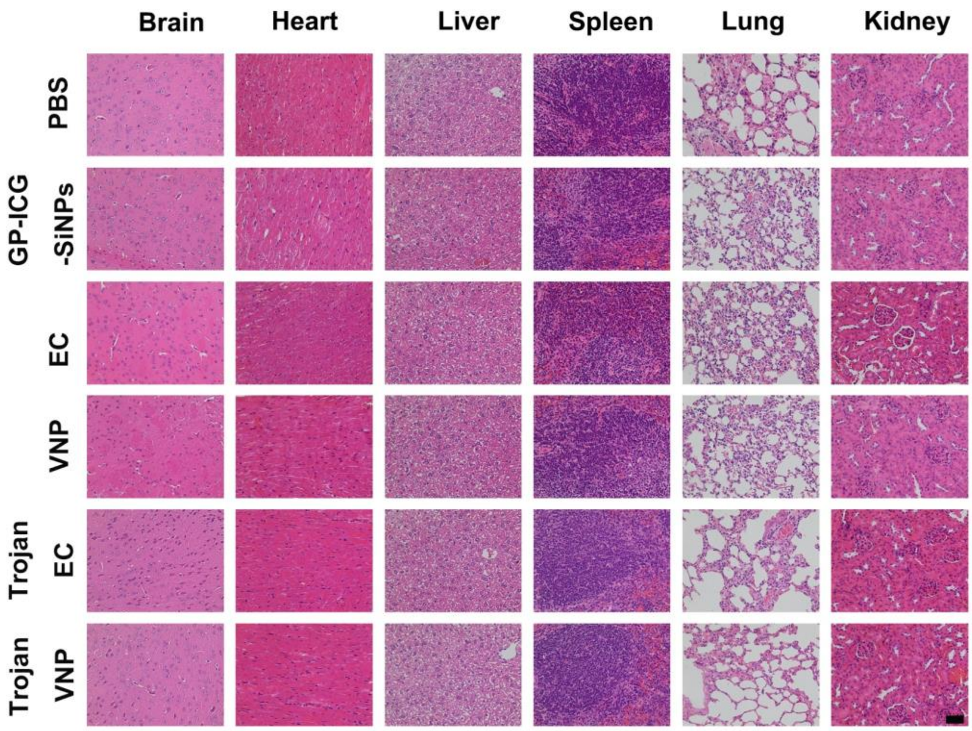

Supplementary Fig. 19. H&E staining of histological evaluation of different organs (brain, heart, liver, spleen, lung and kidney) harvested from the GBM-bearing mice with different treatments. The mice were intravenously injected with PBS, GP-ICG-SiNPs (8 mg/kg ICG), ~1×10^7 CFU EC, ~1×10^7 CFU VNP, Trojan EC (GP-ICG-SiNPs (8 mg/kg ICG) internalized into ~1×10^7 CFU EC) and Trojan VNP (GP-ICG-SiNPs (8 mg/kg ICG) internalized into 1×10^7 CFU VNP), respectively. At the 12-hour post-injection, the brains of those mice were suffered by an 808 nm irradiation (1.2W/cm^2, 5 min). At 30-day post-injection, the main organs were harvested for H&E staining. Scale bars, 50 μm. All imaging experiments were repeated three times with similar results.

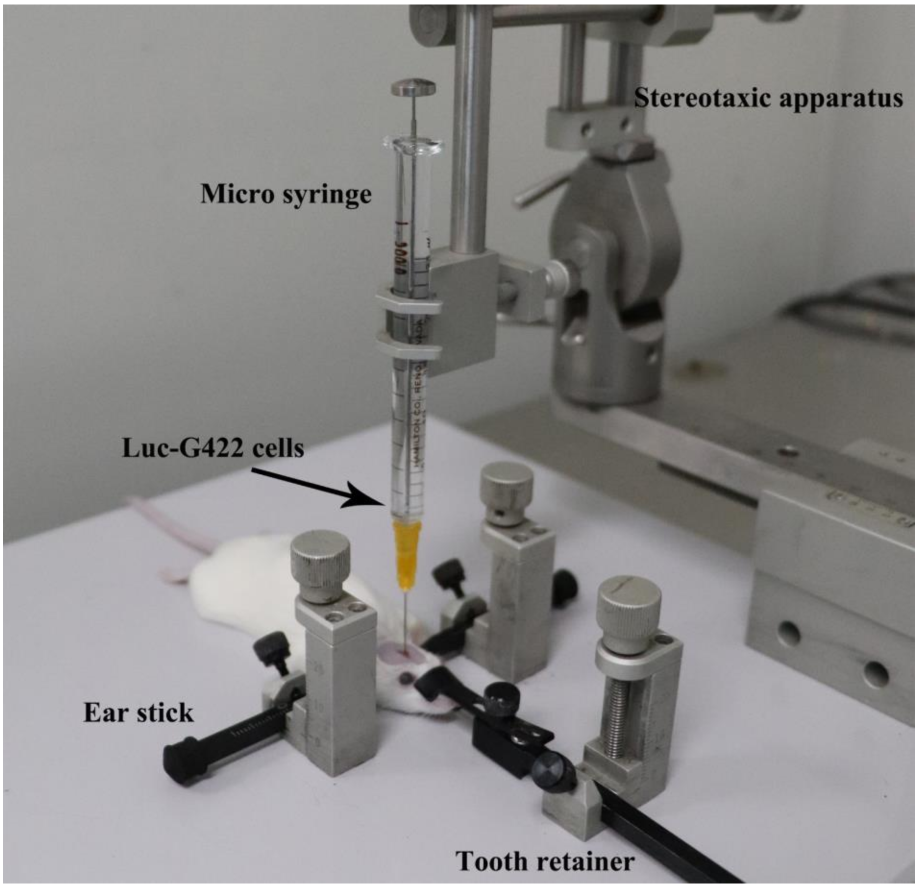

Supplementary Fig. 20 The construction of the orthotopic GBM-bearing mouse model by using the brain stereotaxic apparatus.

Supplementary Table 1. Antibody panel for spectral flow cytometry analyses

Cells	**DC cells**		**CD8$^+$ T cells**				**NK cells**			**Macrophages**	
Marker	**CD11c**	**MHC II**	**CD3**	**CD4**	**CD8**	**CD45**	**CD3**	**NK1.1**	**CD45**	**CD11b**	**F_4/80**
Antigen location	Extra	Extra	Extra	Extra	Intra	Extra	Extra	Extra	Extra	Extra	Extra
Dye	FITC	PerCP	FITC	APC	PE	PerCP	FITC	PE	PerCP	PE	FITC
Ex/Em (nm)	495/ 525	482/ 695	495/ 525	650/ 660	565/ 575	482/ 695	495/ 525	565/ 575	482/ 695	565/ 575	495/ 525
Dilution	1:40	1:40	1:40	1:40	1:40	1:40	1:40	1:40	1:40	1:40	1:40

Four separate panels of flow antibodies were designed for the spectral flow cytometry assays using the flow cytometer (BD Accuri® C6 Plus Flow Cytometry). Extra, Extracellular; Intra, intracellular; Ex/Em, excitation/emission.

Supplementary Table 2. Blood biochemistry and hematology data of healthy mice intravenously injected with EC at the dose of 1.0×10^7 CFU per mouse at 1, 5, and 15 d post injection (n=3).

**Analysis index**	**Normal range**		**EC**					
			**1 day**		**5 day**		**15 day**	
	**Mean**	**Standard deviation**	**Mean**	**Standard deviation**	**Mean**	**Standard deviation**	**Mean**	**Standard deviation**
Albumin and Globulin Ratio	2.41	0.13	2.31	0.11	2.03	0.12	2.46	0.08
White blood cell (10^3 cells/μL)	9.40	1.20	2.82	0.83	8.37	1.89	8.61	1.60
Red blood cell (10^6 cells/μL)	9.12	1.56	7.42	1.24	8.03	2.12	8.41	1.25
Mean corpuscular volume (fL)	51.33	3.67	50.90	3.12	53.67	1.26	51.33	2.70
Mean corpuscular hemoglobin (pg)	13.53	1.90	13.97	1.43	13.47	1.21	13.57	1.12
Mean corpuscular hemoglobin concentration (g/dL)	28.20	3.01	27.93	2.84	27.57	1.68	27.43	1.35
Platelet (10^3 cells/μL)	639.00	109.50	235.67	76.56	807.33	67.83	659.00	85.58
Blood urea nitrogen (mmol/L)	7.61	0.38	5.15	0.23	7.38	0.24	7.67	0.30
Alanine aminotransferase (U/L)	26.64	4.26	48.99	3.81	28.10	3.27	26.25	3.82
Aspartate aminotransferase (U/L)	84.73	15.22	151.55	10.86	103.73	20.68	98.32	2.80
Alkaline phosphatase (U/L)	127.12	7.38	74.79	9.75	125.26	4.34	129.00	8.58

Supplementary Table 3. Blood biochemistry and hematology data of healthy mice intravenously injected with VNP at the dose of 1.0×10^7 CFU per mouse at 1, 5, and 15 d post injection (n=3).

**Analysis index**	**Normal range**		**VNP**					
			**1 day**		**5 day**		**15 day**	
	**Mean**	**Standard deviation**	**Mean**	**Standard deviation**	**Mean**	**Standard deviation**	**Mean**	**Standard deviation**
Albumin and Globulin Ratio	2.54	0.16	2.37	0.10	2.08	0.10	2.42	0.14
White blood cell (10^3 cells/μL)	9.06	1.47	3.58	0.69	8.54	0.73	8.74	1.56
Red blood cell (10^6 cells/μL)	9.33	2.13	8.59	1.05	8.47	0.86	8.65	1.29
Mean corpuscular volume (fL)	52.10	2.40	51.70	2.91	55.07	1.21	51.60	1.97
Mean corpuscular hemoglobin (pg)	14.37	0.86	13.97	1.16	13.93	1.06	14.37	0.74
Mean corpuscular hemoglobin concentration (g/dL)	28.23	1.43	27.27	1.10	28.50	2.01	28.13	2.91
Platelet (10^3 cells/μL)	645.67	107.15	253.33	84.10	779.67	66.01	663.33	63.11
Blood urea nitrogen (mmol/L)	7.96	0.18	5.41	0.22	7.42	0.41	7.95	0.30
Alanine aminotransferase (U/L)	31.65	2.34	57.31	4.56	30.64	2.69	30.46	1.39
Aspartate aminotransferase (U/L)	94.70	12.06	154.57	10.80	105.57	3.49	96.02	7.97
Alkaline phosphatase (U/L)	131.42	3.51	83.16	4.03	129.13	3.69	132.00	4.07

Supplementary Table 4. Routine blood tests of tumour-bearing mice after 16 days of Trojan bacteria injection (~1x10^7 CFU per mouse) (n=3).

Analysis index	Normal range		Trojan EC		Trojan VNP	
	Mean	Standard deviation	Mean	Standard deviation	Mean	Standard deviation
Albumin and Globulin Ratio	2.14	0.25	2.28	0.24	2.30	0.15
White blood cell (10^3 cells/μL)	13.75	2.19	12.04	1.79	11.89	2.33
Red blood cell (10^6 cells/μL)	8.79	0.54	8.78	0.64	8.58	0.39
Hemoglobin (g/dL)	142.76	5.62	134.69	1.79	137.24	4.47
Hematocrit (%)	56.83	1.41	54.87	2.79	55.05	2.44
Mean corpuscular volume (fL)	58.21	2.27	58.54	1.06	59.05	2.41
Mean corpuscular hemoglobin (pg)	15.79	0.28	15.56	0.29	15.83	0.38
Mean corpuscular hemoglobin concentration (g/dL)	30.77	2.72	29.20	2.67	30.92	2.04
Platelet (10^3 cells/μL)	582.36	30.83	618.38	17.01	610.97	18.08
Blood urea nitrogen (mmol/L)	6.54	0.96	7.10	0.62	7.08	0.34

Supplementary Notes:

<u>Table of supplementary notes</u>

1. Confirmation of lamB knockout by Sanger sequencing

2. Confirmation of malE knockout by Sanger sequencing

1. Confirmation of lamB knockout by Sanger sequencing

AAAGCCGTGATGTCCAGGTTGGAGCCAATATGTCGCTGGGTATTCGCCCGG
AACATCTACTGCCGAGTGATATCGCTGACGTCATCCTTGAGGGTGAAGTTC
AGGTCGTCGAGCAACTCGGCAACGAAACTCAAATCCATATCCAGATCCCTT
CCATTCGTCAAAACCTGGTGTACCGCCAGAACGACGTGGTGTTGGTAGAA
GAAGGTGCCACATTCGCTATCGGCCTGCCGCCAGAGCGTTGCCATCTGTTC
CGTGAGGATGGCACTGCATGTCGTCGACTGCATAAGGAGCCGGGCGTTTAA
GCACCCCACAAAACACACAAAGCCTGTCACAGGTGATGTGAAAAAAGAA
AAGCAATGACTCAGGAGATAGATAGCAAAACCTGGGCCGGATAAGGCGTT
TACGCCGCATTCGGCAACCAACGCCTGATGCGACGCTTGCGCGTCTTATCA
GGCCTACAACGGCTGTCAAATGTAGGCCGGATAAGGCGTTTACGCCGCATC
CGGCATAAAAACAGGTTGTCATTATCTGAAAGGGGCGAAAGCCCCTCTGAT
TATCGGGTTTAGCGCGCTATTGCCTGGCTACCGCTGAGCTCCAGATTTTGAG
GTGAAAACAATGAAAATGAATAAAAGTCTCATCGTCCTCTGTTTATCAGCA
GGGTTACTGGCAAGCGCGCCTGGAATTAGCCTTGCCGATGTTAACTACGTA
CCGCAAAACACCAGCGACGCGCCAGCCATTCCATCTGCTGCGCTGCAACA
ACTCACCTGGACACCGGTCGATCAATCT

2. Confirmation of malE knockout by Sanger sequencing

GCTGTACGCTCGCCATGCCCTTCTCCCTTTGTAACAACCTGTCATCGACAGC
AACATTCATGATGGGCTGACTATGCGTCATCAGGAGATGGCTTAAATCCTCC
ACCCCCTGGCTTTTTTATGGGGGAGGAGGCGGGAGGATGAGAACACGGCT
TCTGTGAACTAAACCGAGGTCATGTAAGGAATTTCGTGATGTTGCTTGCAA
AAATCGTGGCGATTTTATGTGCGCATCTCCACATTACCGCCAATTCTGTAAC
AGAGATCACACAAAGCGACGGTGGGGCGTAGGGGCAAGGAGGATGGAAA
GAGGTTGCCGTATAAAGAAACTAGAGTCCGTTTAGGTGTTTTCACGAGCAC
TTCACCAACAAGGACCATAGATTTGCTGTGAAATGCCGGATGCGGCGTGAA
CGCCTTGTCCGGCCTACAAAACCGAAACGTATGTAGGCCTGATAAGACGCG
TCAGCGTCGCATCAGGCAGTTGTTGTCGGATAAGGCGTGAAAGCCTTATCC
GTCCTGGAATGAGGAAGAACCCCATGGATGTCATTAAAAAGAAACATTGGT
GGCAAAGCGACGCGCTGAAATGGTCAGTGCTAGGTCTGCTCGGCCTGCTG
GTGGGTTACCTTGTTGTTTTAATGTACGCACAAGGGGAATACCTGTTCGCCA
TTACCACGCTGATATTGAGTTCAGCGGGGCTGTATATTTTCGCCAATCGTAA
AGCCTACGCCTGGCGCTATGTTTACCCGGGAATGGCTGGAATGGGATTATTC
GTCCTCTTCCCTCTGGTCTGCACCATCGCCATTGCCTTCACCA
