# Supplementary material for: Bacteria loaded with glucose polymer and photosensitive ICG silicon-nanoparticles for glioblastoma photothermal immunotherapy
Source: Nat Commun. 2022 Sep 1;13:5127. doi: 10.1038/s41467-022-32837-5 (PMC9433534; doi:10.1038/s41467-022-32837-5)
Supplement: Supplementary file 3 — Reporting Summary [file 41467_2022_32837_MOESM3_ESM.pdf]

Corresponding author(s): Houyu Wang, Yao He

Last updated by author(s): Aug 18, 2022

Reporting Summary

Nature Portfolio wishes to improve the reproducibility of the work that we publish. This form provides structure for consistency and transparency in reporting. For further information on Nature Portfolio policies, see our Editorial Policies and the Editorial Policy Checklist.

Statistics

For all statistical analyses, confirm that the following items are present in the figure legend, table legend, main text, or Methods section.

n/a	Confirmed	
☐	☒	The exact sample size (n) for each experimental group/condition, given as a discrete number and unit of measurement
☐	☒	A statement on whether measurements were taken from distinct samples or whether the same sample was measured repeatedly
☐	☒	The statistical test(s) used AND whether they are one- or two-sided<br>*Only common tests should be described solely by name; describe more complex techniques in the Methods section.*
☒	☐	A description of all covariates tested
☒	☐	A description of any assumptions or corrections, such as tests of normality and adjustment for multiple comparisons
☐	☒	A full description of the statistical parameters including central tendency (e.g. means) or other basic estimates (e.g. regression coefficient) AND variation (e.g. standard deviation) or associated estimates of uncertainty (e.g. confidence intervals)
☐	☒	For null hypothesis testing, the test statistic (e.g. F, t, r) with confidence intervals, effect sizes, degrees of freedom and P value noted<br>*Give P values as exact values whenever suitable.*
☒	☐	For Bayesian analysis, information on the choice of priors and Markov chain Monte Carlo settings
☒	☐	For hierarchical and complex designs, identification of the appropriate level for tests and full reporting of outcomes
☒	☐	Estimates of effect sizes (e.g. Cohen's d, Pearson's r), indicating how they were calculated

Our web collection on statistics for biologists contains articles on many of the points above.

Software and code

Policy information about availability of computer code

Data collection	Leica Application Suite Advanced Fluorescence Lite<br>BD C6 Plus Flow Cytometry<br>Perkin Elmer Lambda 750<br>Malvern ZETASIZER NANO<br>Thermo NanoDrop 2000C<br>IVIS Lumina III In Vivo Imaging System
Data analysis	Softwares used in analysis include Origin, Graphpad Prism.

For manuscripts utilizing custom algorithms or software that are central to the research but not yet described in published literature, software must be made available to editors and reviewers. We strongly encourage code deposition in a community repository (e.g. GitHub). See the Nature Portfolio guidelines for submitting code & software for further information.

Data

Policy information about availability of data

All manuscripts must include a data availability statement. This statement should provide the following information, where applicable:

- Accession codes, unique identifiers, or web links for publicly available datasets
- A description of any restrictions on data availability
- For clinical datasets or third party data, please ensure that the statement adheres to our policy

The authors declare that the data supporting the findings of this study are available within the article, source data, and its Supplementary Information. Source data underlying Fig. 2f, 2g, Fig. 3a, 3b, 3e, 3g, Fig. 4a, 4c, 4d, 4f, 4g, Fig. 5b, 5d, 5e, 5g, 5h, 5i, 5j, Fig. 6c,6e, 6f, 6g, 6i, 6j, 6k, 6l, 6m, 6n, Fig. 7c,7d, 7g, 7h,

Supplementary Fig. 2c, 2d, 2e, 2f, 2h, 2j, 2k, 2l, Supplementary Fig. 3b, Supplementary Fig. 7a, 7b, 7c, 7d, 7e, 7f, Supplementary Fig. 10a, 10b, Supplementary Fig. 11a, 11b, Supplementary Fig. 12a, 12b, 12c, Supplementary Fig. 13b, 13c, Supplementary Fig. 14b are provided as a Source Data file. Any other data are available from the authors upon reasonable request.

Field-specific reporting

Please select the one below that is the best fit for your research. If you are not sure, read the appropriate sections before making your selection.

[x] Life sciences [ ] Behavioural & social sciences [ ] Ecological, evolutionary & environmental sciences

For a reference copy of the document with all sections, see nature.com/documents/nr-reporting-summary-flat.pdf

Life sciences study design

All studies must disclose on these points even when the disclosure is negative.

Sample size	Group sizes for experiments were chosen on the basis of prior experience and literature precedence, so that sufficient numbers were used to ensure reproducibility and determine standard deviations. The number of animals was at least 3. For each sample, two technical replicates were carried out. If there was 20% or greater variation between technical replicates, an additional two technical replicates were carried out. . Sample sizes employed in this study were referenced previously published studies (Nature Communications 2022, 13: 1255).
Data exclusions	No data were excluded from the analyses.
Replication	All experimental findings were reliably reproduced. At least three independent samples were performed for each experiment. All experiments were performed as technical or biological replications as appropriate for the experiment design. Details of experimental replicates are given in the figure legends
Randomization	Samples were organized into groups according to date of collection. Microorganisms were cultured and maintained in the same environment and randomly allocated to each group.
Blinding	All the data collection and analysis were from blinded with randomized samples.

Reporting for specific materials, systems and methods

We require information from authors about some types of materials, experimental systems and methods used in many studies. Here, indicate whether each material, system or method listed is relevant to your study. If you are not sure if a list item applies to your research, read the appropriate section before selecting a response.

Materials & experimental systems

n/a	Involved in the study	
[ ]	[x]	Antibodies
[ ]	[x]	Eukaryotic cell lines
[x]	[ ]	Palaeontology and archaeology
[ ]	[x]	Animals and other organisms
[ ]	[x]	Human research participants
[x]	[ ]	Clinical data
[x]	[ ]	Dual use research of concern

Methods

n/a	Involved in the study	
[x]	[ ]	ChIP-seq
[ ]	[x]	Flow cytometry
[x]	[ ]	MRI-based neuroimaging

Antibodies

Antibodies used	Antibodies for flow cytometry are described in detail in Supplementary Table 1. All other antibodies were described in detail in Materials and Methods. The details of antibodies are as follow, anti-CD11c-FITC (BioLegend, clone: N418, catalog no. 117306, lot: B331570),anti-MHCII-PerCP(Elabscience, clone: M5/114 ,catalog no. E-AB-F0990F, lot:212271） anti-CD45-PercP(Bioscience, clone: 30-F11,catalog no. E-AB-F1136F, lot:225050), anti–CD3-FITC (BioLegend, clone: 145-2C11, catalog no. 100306, lot: B241616), anti-CD4-APC (BioLegend, clone: GK1.5, catalog no. 100411, lot: B332955), anti-CD8a-PE(Elabscience, clone: 53-6.7, catalog no. E-AB-F11041, lot: 224571), anti-NK1.1-PE(Elabscience,clone: PK136,catalog no. E-AB-F0987D, lot:225122), anti-F4/80-FITC (Biolegend,clone:BM8, catalog no. 123107,lot:B320936), anti-CD11b-PE（Biolegend, clone:M1/70,catalog no. 101207,lot:B323633), anti-HIF-1α (Merck, clone: H1α67, catalog no. MAB5382).
Validation	All primary antibodies were mouse-specific as per the company's data sheet. All used antibodies have been previously published and the references can be found on data sheets

Eukaryotic cell lines

Policy information about cell lines

Cell line source(s)	HBMEC, bEnd.3, U87MG, 4T1, GL261, HeLa, G422 and (Luc)-G422 cells were sourced form Shanghai Zhong Qiao Xin Zhou Biotechnology Co., Ltd (China)
Authentication	Cell lines were used from the source without authentication.
Mycoplasma contamination	Cells were tested monthly and found to be negative for mycoplasma contamination.
Commonly misidentified lines (See ICLAC register)	No commonly misidentified cell lines were used.

Animals and other organisms

Policy information about studies involving animals; ARRIVE guidelines recommended for reporting animal research

Laboratory animals	The housing conditions for the mice (female, 10-12 weeks old) were 25 °C and 65% humidity adjusted by the ventilation equipment and air filtration system. All animals are provided with 12 h of light and 12 h of darkness daily. Weekly cleaning of the cages and changing of the corn cob bedding are carried out by specialized staff. The animals are fed with irradiated feed and water, and the animals are fed with autoclaved tap water.
Wild animals	no wild animals were used in the study.
Field-collected samples	no field collected samples were used in the study.
Ethics oversight	all animal experimental procedures were performed according to the Guideline for Animal Experimentation with the approval of the animal care committee of Soochow University.

Note that full information on the approval of the study protocol must also be provided in the manuscript.

Human research participants

Policy information about studies involving human research participants

Population characteristics	Human blood samples were provided by a healthy volunteer.
Recruitment	Human blood samples were provided by a healthy volunteer following written informed consent.
Ethics oversight	The study protocols using human blood samples were approved by the ethics committee of Soochow University. The authors state that all human blood experiments were performed in strict accordance with the relevant laws and institutional guidelines.

Note that full information on the approval of the study protocol must also be provided in the manuscript.

Flow Cytometry

Plots

Confirm that:

- [x] The axis labels state the marker and fluorochrome used (e.g. CD4-FITC).
- [x] The axis scales are clearly visible. Include numbers along axes only for bottom left plot of group (a 'group' is an analysis of identical markers).
- [x] All plots are contour plots with outliers or pseudocolor plots.
- [x] A numerical value for number of cells or percentage (with statistics) is provided.

Methodology

Sample preparation	Tumors, spleen and the carotid lymph nodes were harvested from sacrificed mice. The tumors and lymph nodes were cut into small pieces and resuspended in DMEM. The supernatant from the clipped lymph nodes was collected, centrifuged at 490 x g for 5 min, and resuspended. The tumour pieces were incubated with digestive enzyme for 1 h at 37°C on a shaker (90 rpm) and then filtered through a cell strainer. The supernatant from the digested tumor tissues was collected, centrifuged at 490 x g for 5 min, and resuspended. The spleen was mechanically dissociated and resuspended in DMEM. The suspension was filtered through a cell strainer, centrifuged and resuspended. Lymphocytes were stained with anti-CD11c-FITC, anti- MHC II-PerCP for DC maturation analysis, while the single tumour cell suspensions labeled with anti-CD45-PercP, anti-CD3-FITC, anti-CD4-APC and anti-CD8a-PE was used to examine CD8a+ T cell response. And single tumour cell suspensions labeled with labeled with anti-CD45-PercP, anti-CD3-FITC, anti-NK1.1-PE was used to examine NK cell response. Meanwhile to examine macrophage cell response, single tumour cell suspensions were labeled with anti-CD11b-PE, anti-F4/80-FITC. The cells were then washed twice and analysed using flow cytometer.

Instrument	BD C6 Plus Flow Cytometry
Software	FlowJo_V10
Cell population abundance	DCs were used as the only cell population.
Gating strategy	Initial cell populations were gated for a live population using FSC and SSC plot of cell only sample. The gate was set to removecell debris and dead cells (small FSC and SSC) and large clumps or aggregates of cells (large FSC and SSC) and used across allsamples. This live population was then further gated .

☒ Tick this box to confirm that a figure exemplifying the gating strategy is provided in the Supplementary Information.
